# The Activity of Class I, II, III and IV of Alcohol Dehydrogenase (ADH) Isoenzymes and Aldehyde Dehydrogenase (ALDH) in Brain Cancer

**DOI:** 10.1007/s11064-013-1053-9

**Published:** 2013-04-27

**Authors:** Magdalena Laniewska-Dunaj, Wojciech Jelski, Karolina Orywal, Jan Kochanowicz, Robert Rutkowski, Maciej Szmitkowski

**Affiliations:** 1 Department of Biochemical Diagnostics, Medical University, Sklodowskiej-Curie 24 A, 15–276 Bialystok, Poland; 2 Department of Neurosurgery, Medical University, Bialystok, Poland

**Keywords:** Alcohol dehydrogenase isoenzymes, Aldehyde dehydrogenase, Brain cancer

## Abstract

The brain being highly sensitive to the action of alcohol is potentially susceptible to its carcinogenic effects. Alcohol dehydrogenase (ADH) and aldehyde dehydrogenase (ALDH) are the main enzymes involved in ethanol metabolism, which leads to the generation of carcinogenic acetaldehyde. Human brain tissue contains various ADH isoenzymes and possess also ALDH activity. The purpose of this study was to compare the capacity for ethanol metabolism measured by ADH isoenzymes and ALDH activity in cancer tissues and healthy brain cells. The samples were taken from 62 brain cancer patients (36 glioblastoma, 26 meningioma). For the measurement of the activity of class I and II ADH isoenzymes and ALDH activity, the fluorometric methods were used. The total ADH activity and activity of class III and IV isoenzymes were measured by the photometric method. The total activity of ADH, and activity of class I ADH were significantly higher in cancer cells than in healthy tissues. The other tested classes of ADH and ALDH did not show statistically significant differences of activity in cancer and in normal cells. Analysis of the enzymes activity did not show significant differences depending on the location of the tumor. The differences in the activity of total alcohol dehydrogenase, and class I isoenzyme between cancer tissues and healthy brain cells might be a factor for metabolic changes and disturbances in low mature cancer cells and additionally might be a reason for higher level of acetaldehyde which can intensify the carcinogenesis.

## Introduction

Human brain tumors are a heterogenous group of neoplasms occurring inside the cranium and the central spinal cord and after stroke, they are the second cause of death from neurological diseases. They are the most common and the most aggressive histological subtype of cranial tumor with a 5-year survival of less than 5 % [1]. The causes of brain tumors are poorly understood. Some studies suggests that low to moderate alcohol consumption might alter brain function and decrease brain health [2]. The mechanisms responsible for these effects are still unknown. Alcohol is capable of traversing the blood–brain barrier and is thus a possible risk factor for brain cancer besides being an established cause for some other cancers and diseases [3]. It is postulated that many neurochemical and neurotoxic effects of ethanol are mediated by the first metabolite of its metabolism-acetaldehyde [4]. Oxidative metabolism of alcohol in human body is mainly catalyzed by alcohol dehydrogenase (ADH) and aldehyde dehydrogenase (ALDH). Human brain contains mainly three classes of alcohol dehydrogenase (ADH) isoenzymes: I, III and IV [5]. These isoenzymes of ADH participate also in the metabolism of many biological substances such as retinol or serotonin. ADH isoenzymes are responsible for the metabolism of ethanol but it has not been definitively demonstrated to play a significant role in brain. Aldehyde dehydrogenase plays the crucial role in the further oxidation of ethanol-derived acetaldehyde in brain cells [6].

The goal of the present study was to compare the capacity for ethanol metabolism measured by ADH isoenzymes (class I, II, III and IV) and ALDH activity in cancer tissues and healthy brain cells.

## Materials and Methods

### Patients

The protocol was approved by the Human Care Committee of the Medical University in Bialystok, Poland (Approval Nr R-I-002/24/2010). All patients gave informed consent for the examination.

Biopsy specimens of brain healthy tissues and cancer tissues (80–100 mg) were taken at the same time during routine surgical operation for treatment of brain tumors of 62 patients (35 males and 27 females, range 30–77 years). All tumors patients were identified from January 2011–December 2012 in Department of Neurosurgery Medical University of Bialystok (Poland). Among the cancer patients, 36 patients (21 men and 15 women) suffering from glioblastoma and 26 patients (14 men, 12 women) had meningioma. The tumors were histopathologically classified according to World Health Organization criteria. We have created the following groups, depending on the location of the tumor: the frontal lobe, temporal lobe, parietal lobe, cerebellum. Classification was based on preoperative computed tomography (CT) and magnetic resonance imaging scans. Preoperative functional status of the patient was evaluated with the Karnofsky Performance Status Scale. Preoperative MRI was used to determine tumor volumes. All of the patients drank alcohol occasionally and self-reported an intake of <25 g of ethanol per week. The clinicopathological characteristics of the patients are shown in Table 1.

**Table 1:** Characteristics of brain cancer patients

Variable		No of patients
Brain cancer patients		62
Gender		
Males		37
Females		27
Age		
<50 years		34
≥50 years		28
Range		30–77
Histological type		
Glioblastoma		36
Meningioma		26
Tumour stage		
I		16
II		10
III		15
IV		21
Tumor size		
<3 cm		35
≥3 cm		27
Location in the brain		
Frontal lobe		16
Temporal lobe		24
Parietal lobe		16
Cerebellum		6
Degree of differentiation		
Well		39
Poorly		23

### Biochemical Assays

#### Determination of Total ADH Activity

Total ADH activity was estimated by the photometric method with *p*-nitrosodimethylaniline (NDMA) as a substrate [7]. The reaction mixture (2 ml) contained 1.9 ml of a 26 μM solution of substrate in 0.1 M of sodium phosphate buffer, pH 8.5 and 0.1 ml of a mixture containing 0.25 M n-butanol and 5 mM NAD. The reduction of NDMA was monitored at 440 nm on a Shimadzu UV/VIS 1202 spectrophotometer (Shimadzu Europa GmbH, Duisburg, Germany).

#### Determination of Total ALDH Activity

ALDH activity was measured using the fluorogenic method based on the oxidation of 6-methoxy-2-naphtaldehyde to the fluorescent 6-methoxy-2 naphtoate [8]. The reaction mixture contained 60 μl of substrate, 20 μl of 11.4 mM NAD and 2.8 ml of 50 mM of sodium phosphate buffer, pH 8.5. The mixture contained also 50 μl of a 12 mM solution of 4-methylpyrazole as a specific inhibitor of ADH activity. The fluorescence was read at excitation wavelength 310 and emission wavelength 360 nm.

#### Determination of Class I and II ADH Isoenzymes

Fluorometric assays of alcohol dehydrogenase isoenzyme activities (class I and II) are more sensitive and specific than the previously used classical method. Class I and II alcohol dehydrogenase isoenzyme activity were measured using fluorogenic substrates (4-methoxy-1-naphthaldehyde for class I and 6-methoxy-2-naphthaldehyde for class II) in reduction reaction according to Wierzchowski et al. [9]. The assays were performed in a reaction mixture containing a serum (60 μl), substrate (150 μl of 300 μM), NADH (100 μl of 1 mM) and 0.1 M of sodium phosphate buffer, pH 7.6 (2.69 ml) in conditions previously described [10]. The measurements were performed on a Shimadzu RF–5301 spectrofluorophotometer (Shimadzu Europa GmbH, Duisburg, Germany) at excitation wavelenght 316 nm for both substrates and emission of 370 nm for class I and 360 nm for class II isoenzymes.

#### Determination of Class III ADH Isoenzyme

The assay mixture for class III of alcohol dehydrogenase activity contained a serum (100 μl), *n*–octanol as a substrate (31 μl of 1 mM), NAD (240 μl of 1.2 mM) in 0.1 M NaOH-glycine buffer pH of 9.6 [11]. The reduction of NAD was monitored at 340 nm and 25 °C on a Shimadzu UV/VIS 1202 spectrophotometer.

#### Determination of Class IV ADH Isoenzyme

The assay mixture for class IV of alcohol dehydrogenase activity contained a serum (50 μl), m–nitrobenzaldehyde as a substrate of (132 μl of 80 μM), NADH (172 μl of 86 μM) in 0.1 M sodium phosphate buffer pH 7.5 [12]. The oxidation of NADH was monitored at 340 nm and 25 °C on a Shimadzu UV/VIS 1202 spectrophotometer.

### Statistical Analysis

A preliminary statistical analysis (Chi square test) revealed that the distribution of ADH and ALDH activities did not follow a normal distribution. Consequently, the Wilcoxon’s test was used for statistical analysis. Data were presented as median, range and mean values. Statistically significant differences were defined as comparisons resulting in *p* < 0.05.

## Results

The activities of total ADH, ALDH and ADH isoenzymes in brain tumor cells are demonstrated in Table 2. We have shown that ADH and ALDH activities are present in cancer cells, although ALDH activity is 3 times lower than ADH in all patients groups. Measuring the activity of ADH isoenzymes found that the median activity of class III was 0.552 nmol/min/mg of protein in cancer and 0.513 nmol/min/mg of protein in healthy tissue in total patients group. The activity of class I ADH in cancer and normal cells was 0.431 nmol/min/mg of protein and 0.346 nmol/min/mg of protein respectively. The activity of ADH IV was 0.363 nmol/min/mg of protein in cancer and 0.332 nmol/min/mg of protein in healthy tissue. The activity of class II ADH isoenzyme is barely detectable.

**Table 2:** The activity of ADH isoenzymes and ALDH in brain cancer tissue

Tested group	Adh I Median Range Mean	ADH II Median Range Mean	ADH III Median Range Mean	ADH IV Median Range Mean	ADH total Median Range Mean	ALDH Median Range Mean
Brain cancer (n = 62)	0.431	0.0029	0.552	0.363	2.485	0.808
	0.203–0.722	0.0008–0.0074	0.175–1.322	0.152–0.734	1.164–6.732	0.396–2.015
	0.422	0.0028	0.535	0.344	2.378	0.783
Glioblastoma (n = 36)	0.406	0.0030	0.575	0.370	2.531	0.813
	0.216–0.722	0.0010–0.0074	0.198–1.332	0.177–0.734	1.302–6.732	0.406–2.105
	0.454	0.0029	0.561	0.362	2.428	0.798
Meningioma (n = 26)	0.426	0.0028	0.546	0.361	2.453	0.804
	0.203–0.684	0.0008–0.0069	0.175–1.248	0.152–0.688	1.164–5.975	0.396–1.846
	0.417	0.0028	0.527	0.339	2.334	0.776
Healthy tissue (n = 62)	0.346	0.0028	0.513	0.332	1.798	0.746
	0.165–0.606	0.0006–0.0065	0.134–1.215	0.138–0.685	0.955–5.012	0.266–1.984
	0.341	0.0027	0.495	0.311	1.638	0.727
	p < 0.001^a^	p = 0.276^a^	p = 0.373^a^	p = 0.387^a^	p < 0.001^a^	p = 0.389^a^
	p < 0.001^b^	p = 0.357^b^	p = 0.304^b^	p = 0.387^b^	p < 0.001^b^	p = 0.428^b^
	p < 0.001^c^	p = 0.309^c^	p = 0.327^c^	p = 0.303^c^	p < 0.001^c^	p = 0.327^c^
	p = 0.365^d^	p = 0.574^d^	p = 0.429^d^	p = 0.329^d^	p = 0.538^d^	p = 0.377^d^

Data are expressed as nmol/min/mg of protein

^a^Brain cancer versus healthy tissue

^b^Glioblastoma versus healthy tissue

^c^Meningioma versus healthy tissue

^d^Glioblastoma versus meningioma

We found that the activity of class I of ADH is significantly higher (*p* < 0.001) in brain cancer than in healthy tissue in total patients group and in both, glioblastoma and meningioma. The other tested classes of ADH and ALDH did not show statistically significant differences of activity in cancer and in normal cells (*p* > 0.05). The total activity of alcohol dehydrogenase was significantly higher in brain cancer than in healthy ones (about 28 %). The median activity of total ADH was 2.485 nmol/min/mg of protein in cancer and 1.798 nmol/min/mg of protein in healthy tissue in total patients group. The median activity of total ALDH was 0.808 nmol/min/mg of protein in cancer and 0.746 nmol/min/mg of protein in healthy tissue.

The analysis of the enzymes and isoenzymes activities did not indicate significant differences between glioblastoma and meningioma.

**Fig. 1:**
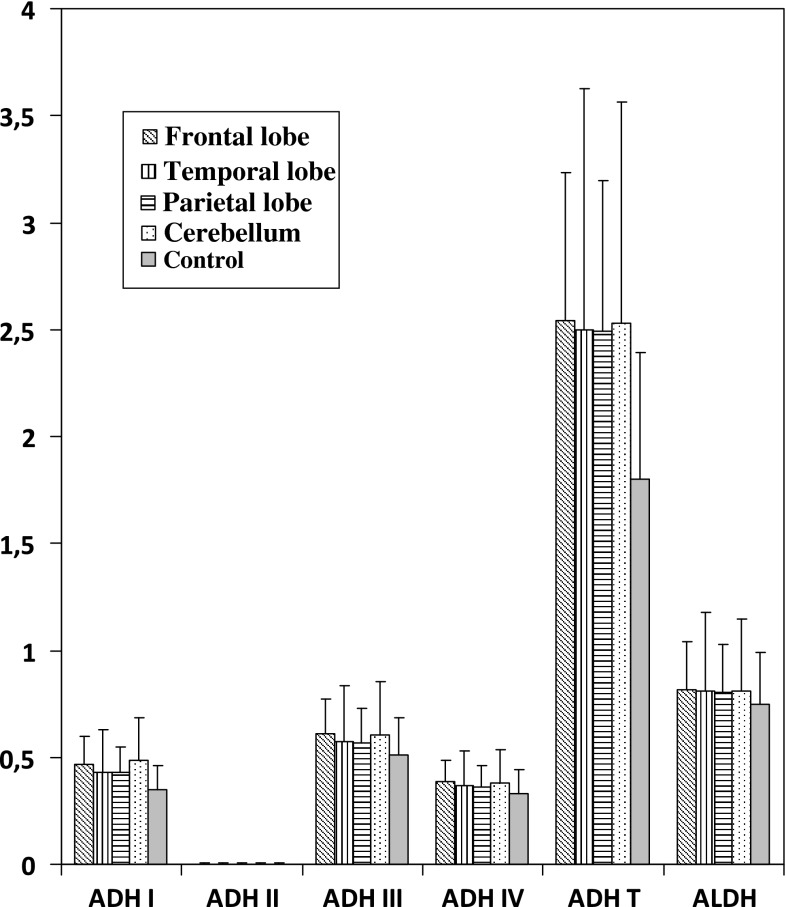
The comparison of ADH isoenzymes and ALDH activity in cancer tissue depending on the localization of tumour. Data are expressed as a median in nmol/min/mg of protein

The analysis of ADH, ALDH and ADH isoenzymes activities showed lack of statistically significant difference depending on the location of the tumor (Fig. 1).

## Discussion

Brain cancer accounts for approximately 1.4 % of all cancers and is responsible for about 2.3 % of all cancer-related deaths. Glioblastoma and meningioma are the most common subtypes, accounting for over 80 % of brain tumor cases [13]. Although there has been a recent increase in the number of epidemiologic studies of brain cancer, little consensus exists regarding the nature and magnitude of the risk factors contributing to its development. Some studies suggest that alcohol consumption increases the risk of brain tumor consistent with a dose–response relationship [14, 15]. Alcohol could exert a carcinogenic effect in the brain directly by altering the expression of genes but, the most important ways are probably related to alcohol mechanism. It is postulated that many neurotoxic effects of ethanol are mediated by the first metabolite, acetaldehyde [16]. This requires the presence of acetaldehyde inside the brain. The possibility of acetaldehyde appearance inside the brain is the production during the ethanol oxidation in situ. Zimatkin and co-workers found that the catalase and cytochrome P-450-dependent system plays an important role (about 80 %) in ethanol oxidation in the brain. The rest of the ethanol-oxidizing activity of the brain is due to alcohol dehydrogenase [6]. The involvement of ADH in ethanol oxidation in the brain cannot be excluded because of the data concerning the translation of ADH I in the brain tissue [17]. Acetaldehyde formed in the brain is then oxidized by aldehyde dehydrogenase [6]. However in our study we found that the activity of ADH was significantly higher in cancer tissues than that in healthy and the activity of ALDH was not different between both tissues. It is important that activity of ADH is higher than the activity of ALDH. The levels of acetaldehyde are dependent on the balance of activity of these enzymes.The activity of ADH and ALDH is in the ratio of 3: 1 in cancer cells and only 2:1 in healthy brain tissue. This would suggest that the cancer cells have a big capability to ethanol oxidation to acetaldehyde and small capability to remove acetaldehyde. Moreover in our study we have found that the activity of class I ADH in brain cancer (0.431 nmol/min/mg of protein) is about 20 % higher than in normal tissue (0.346 nmol/min/mg of protein). The main ethanol-metabolizing isoenzyme in the brain is class I of ADH. The metabolism of ethanol by ADH I leads to generation of acetaldehyde, which is cytotoxic, is implicated in ethanol-induced cell damage and is carcinogenic for humans. Our study demonstrates that the highest activity of all tested ADH isoenzymes exhibited the isoenzyme of class III. However the kinetic properties of human class III ADH isoenzyme indicates that this isoenzyme cannot be saturated by ethanol even at high concentration [18]. The Michaelis constant of ADH III for ethanol is higher than 3,000 mmol/l and ADH III has a very low affinity for this alcohol as a substrate. Therefore the human class III alcohol dehydrogenase isoenzyme cannot oxidize ethanol in brain, because so high concentration is not found in this organ. As the substrates of this isoenzyme include intrinsic toxic formaldehyde, inflammatory intermediate 20-hydroxy-leukotriene. The distribution of class III ADH immunostaining, indicates that this enzyme contributes to defence of the brain against degenerative processes [19].

Our study demonstrates that activity of class II ADH is barely detectable in cancer cells and healthy brain tissue. It is in agreement with data reported by Martinez et al. Martinez and colleagues have investigated isoenzymes of ADH in the rat brain tissue and found no expression of ADH II mRNA in tested brain regions [18].

The histological differences of cancers did not effect on the activity of tested enzymes.We have found that the activity of ADH and ALDH in cancer tissues of glioblastoma did not differ from that of meningioma. It is interesting because they differ in grade of malignancy, origin of cells and etiology.

Malignancy of brain cancer may be due not only to its histological subtype, and the origin of the cells but also to the location of the tumor. The value of this study was the investigation of the possibility of ADH activity relationships to a variety of tumor locations. Analyzing the activity of ADH, ALDH and ADH isoenzymes we do not find the significant differences depending on the location of the tumor in the brain. Although it has been shown that the activity of individual isoenzymes of ADH in tumor tissue correlate with their occurrence in specific brain regions. The high activity of ADH III was found in tumors located in the cerebellum and frontal lobe. Healthy brain tissue in these regions is also characterized by high activity of ADH III [5, 20]. Similar observations apply to the class I ADH isoenzyme, which besides ADH III, is the major isoenzyme of alcohol dehydrogenase present in the brain.

In conclusion we can state that brain cancer exhibites ADH and ALDH activities. Among the ADH isoenzymes, class I ADH showes statistically significant difference between cancerous tissue and healthy brain cells. The increased activity of class I ADH in brain cancer cells suggests the increased ability to ethanol metabolism and formation of acetaldehyde which is metabolized by normal activity of ALDH. This may increase its concentration and intensify the carcinogenesis. Moreover ADH/ALDH is 3 in cancer cells and only 2 in healthy brain tissue, suggesting cancer cells are more prone to ethanol oxidation but less to removal of acetaldehyde.
